# Emergency Closed Reduction of a C4/5 Fracture Dislocation with Complete Paraplegia Resulting in Profound Neurologic Recovery

**DOI:** 10.1155/2013/272865

**Published:** 2013-09-12

**Authors:** Christian W. Müller, Sebastian Decker, Roland Thietje, Christian Krettek

**Affiliations:** ^1^Trauma Department, Hannover Medical School (MHH), Carl-Neuberg Street 1, 30625 Hannover, Germany; ^2^Centre for Spinal Injuries, BG Trauma Hospital Hamburg, Bergedorfer Straße 10, 21033 Hamburg, Germany

## Abstract

*Introduction*. Cervical spinal cord injuries due to traumatic fractures are associated with persistent neurological deficits. Although clinical evidence is weak, early decompression, defined as <24–72 h, has been frequently proposed. Animal studies show better outcomes after early decompression within one hour or less, which can hardly ever be achieved in clinical practice. *Case Presentation*. A 37-year-old patient was hospitalized after being hit by a shying horse. After diagnosis of C4/5 fracture dislocation and complete paraplegia, she was intubated and sedated with deep relaxation. Emergency reduction was performed at approximately 120 minutes after trauma. Subsequently, a standard anterior decompression, discectomy, and fusion were carried out. She was then transferred to a specialized rehabilitation hospital. Her neurologic function improved from AIS grade A on admission to grade B postoperatively and grade D after four months of rehabilitation. One year after the accident, she was ambulatory without walking aids and restarted horse riding. *Discussion and Conclusion*. Rarely in clinical practice, decompression of the spine canal can be performed as early as in this case. This case highlights the potential benefit of utmost early reduction in cervical fracture dislocations with compression of the spinal cord.

## 1. Introduction

Spinal cord injuries due to traumatic fractures of the cervical spine can be associated with persistent neurological deficits. However, clinical options to improve spinal cord function are sparse [1]. Conflicting data exist as to the benefit of high dose methylprednisolone therapy in cervical spinal cord injury. A recent prospective trial showed a nonsignificant better neurologic recovery in the group *without* methylprednisolone therapy [2], while other researchers reported higher complication rates following methylprednisolone therapy [3].

Although clinical evidence is weak, early decompression, defined as <24–72 h, has been frequently proposed [4, 5]. Animal studies clearly showed better outcomes after early decompression within one hour or less, which can hardly ever be achieved in clinical practice [5]. In AIS grade B patients, McCarthy et al. found a higher rate of improvement of at least one grade when decompression was performed in less than eight hours after injury [6].

Hitherto, no data on emergency closed reduction prior to surgery exists. Yisheng et al. reported on a series of 290 patients suffering from cervical spinal cord injuries with fracture and dislocation. They concluded that first aid measures of early closed reduction or realignment and immobilization of the cervical spine, breathing support, and high-dose methylprednisolone were the most important in the treatment for traumatic spinal cord injury. However, while the majority of the patients in their series with AIS grade C neurological deficit (102/124) regained the ability to walk postoperatively, most of the seriously injured patients (AIS grades A and B) had no improvement of their neurological function [7]. Marino et al., though, found that 24 out of 336 patients with AIS grade A improved to grade D at one-year followup [8].

## 2. Case Presentation

**Figure 1:**
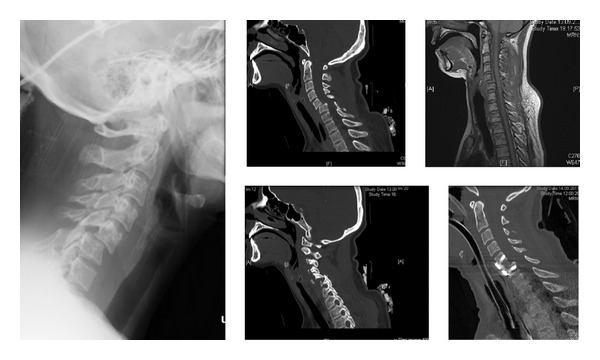
Conventional X-rays on admission showing fracture dislocation C4/5 (left); preoperative CT scan showing unilateral facet dislocation (middle); MRI after closed reduction (upper right); CT scan after anterior decompression and stabilization (lower right).

A 37-year-old patient was hospitalized after being hit by a shying horse. Rescue personnel documented complete paraplegia of both legs. She was admitted to a level I trauma center. On arrival, the patient was awake with no motor function of the legs and impaired motor function of the arms; motor and sensory levels of sub C7 were determined with partial remaining sensation up to Th10 (American Spinal Injury Association Impairment Scale (AIS) grade A). Along with the ATLS guidelines, a lateral conventional X-ray of the cervical spine was conducted, which showed fracture dislocation C4/5 (cf.  Figure 1, left). According to the NASCIS-2 protocol, high-dose methylprednisolone was administered [9]. Immediate trauma CT scan showed fracture dislocation C4/5 with partially locked facet dislocation C4/5 on the right side and raised suspicion for dissection of the right vertebral artery (cf.  Figure 1, middle). She was then intubated and sedated with deep relaxation. Emergency reduction was performed at approximately 120 minutes after trauma, and MRI scans were performed, which showed a marked reduction and confirmed dissection of both right vertebral and internal carotid arteries (cf.  Figure 1, upper right). Subsequently, at approximately 7 hours after trauma, a standard anterior decompression, discectomy, and fusion were carried out (cf.  Figure 1, lower right). Anticoagulation therapy and physiotherapy were started the day after. Postoperatively, there was an improvement of sensation in both legs, but no change in motor function of the legs (AIS grade B). After one week, she was transferred to a specialized rehabilitation hospital. Within 4 months of intensive physiotherapy and occupational therapy, she regained normal bladder and bowel functions. One year after the accident, she was ambulatory without walking aids and restarted horse riding (AIS grade D).

## 3. Discussion and Conclusion

Rarely in clinical practice, decompression of the spine canal can be performed as early as in this case. Here, a surprisingly good clinical result with almost full recovery in all aspects was achieved. Yet, the risk of further harm due to potential posterior dislocation of the ruptured disk has to be pondered when attempting closed reduction of the dislocated cervical spine. 

This case highlights the potential benefit of the concept of utmost early reduction in fracture dislocations with compression of the spinal cord, followed by early definitive stabilization and intensive neurological rehabilitation.
